# Characterization of post-edited cells modified in the *TFAM* gene by CRISPR/Cas9 technology in the bovine model

**DOI:** 10.1371/journal.pone.0235856

**Published:** 2020-07-10

**Authors:** Vanessa Cristina de Oliveira, Clésio Gomes Mariano Junior, José Ernesto Belizário, José Eduardo Krieger, Fabiana Fernandes Bressan, Kelly Cristine Santos Roballo, Paulo Fantinato-Neto, Flávio Vieira Meirelles, Marcos Roberto Chiaratti, Jean-Paul Concordet, Carlos Eduardo Ambrósio

**Affiliations:** 1 Department of Veterinary Medicine, Faculty of Animal Science and Food Engineering, University of São Paulo, Pirassununga, São Paulo, Brazil; 2 Department of Pharmacology, Institute of Biomedical Sciences, University of São Paulo, São Paulo, Brazil; 3 Laboratory of Genetics and Molecular Cardiology, Heart Institute, University of São Paulo Medical School, São Paulo, Brazil; 4 School of Pharmacy at College of Health Sciences, University of Wyoming, Laramie, WY, United States of America; 5 Department of Genetics and Evolution, Federal University of São Carlos, São Carlos, SP, Brazil; 6 Laboratoire Structure et Instabilité des Génomes, Museum National d’Histoire Naturelle, INSERM U1154, CNRS UMR7196, Paris, France; University of Florida, UNITED STATES

## Abstract

Gene editing in large animal models for future applications in translational medicine and food production must be deeply investigated for an increase of knowledge. The mitochondrial transcription factor A (*TFAM*) is a member of the HMGB subfamily that binds to mtDNA promoters. This gene maintains mtDNA, and it is essential for the initiation of mtDNA transcription. Lately, we generated a new cell line through the disruption of the *TFAM* gene in bovine fibroblast cells by CRISPR/Cas 9 technology. We showed that the CRISPR/Cas9 design was efficient through the generation of heterozygous mutant clones. In this context, once this gene regulates the mtDNA replication specificity, the study aimed to determine if the post-edited cells are capable of *in vitro* maintenance and assess if they present changes in mtDNA copies and mitochondrial membrane potential after successive passages in culture. The post-edited cells were expanded in culture, and we performed a growth curve, doubling time, cell viability, mitochondrial DNA copy number, and mitochondrial membrane potential assays. The editing process did not make cell culture unfeasible, even though cell growth rate and viability were decreased compared to control since we observed the cells grow well when cultured in a medium supplemented with uridine and pyruvate. They also exhibited a classical fibroblastoid appearance. The RT-qPCR to determine the mtDNA copy number showed a decrease in the edited clones compared to the non-edited ones (control) in different cell passages. Cell staining with Mitotracker Green and red suggests a reduction in red fluorescence in the edited cells compared to the non-edited cells. Thus, through characterization, we demonstrated that the *TFAM* gene is critical to mitochondrial maintenance due to its interference in the stability of the mitochondrial DNA copy number in different cell passages and membrane potential confirming the decrease in mitochondrial activity in cells edited in heterozygosis.

## Introduction

Mitochondria are intracellular organelles in charge of ATP synthesis, and they have their sole genetic material, the mitochondrial DNA (mtDNA) [1, 2].

The mtDNA is a circular double-stranded molecule essential for mitochondrial functioning and, consequently, for cellular performance. It encodes several subunits of the proteins in the electron transport chain and transporter and ribosomal RNAs [3]. It is replicated independently of nuclear DNA (nDNA), which codes the proteins responsible for mtDNA replication [4].

Among these various proteins, the mitochondrial transcription factor A (*TFAM*) is a protein that, after being encoded in the nucleus and translated it is exported to the mitochondria. In several studies, *TFAM* has been considered the main protein in this process due to its architectural role in mtDNA packaging in nucleoids. It also acts in the replication, transcription, and regulation of the number of copies of mtDNA [5–7].

Like nDNA, mtDNA is subject to damage from other molecules, both exogenous and endogenous [8]. Among the endogenous, the reactive oxygen species (ROS) may be the main cause of damage and mutations, with potentially pathological action on mtDNA [9]. The *TFAM* function in mtDNA compacting is similar to the role of nuclear histones in the organization of chromatin. Therefore, it is essential for the conservation of mitochondrial genetic material and protection against possible pathological mutations [6].

Several diseases are related to mitochondrial disorders such as Parkinson's, Huntington's Disease, Alzheimer's, Frontotemporal Dementia, Amyotrophic Lateral Sclerosis, and peripheral neuropathies linked to Diabetes [10–13]. These are different but very similar when analyzed molecularly, which opens the door to a unique treatment of mitochondrial origin that can reduce or even reverse the symptoms of them [14,15].

In this sense, recent advances in genome editing techniques have made it possible to modify any desired DNA sequence using programmable nucleases. Gene editing tools using the CRISPR/Cas mechanism (Clustered Regularly Interspaced Short Palindromic Repeats) are the most widely used and effective.

The CRISPR/Cas9 technology is a relatively simple, accurate, and efficient tool that has emerged from the study of bacteria. The CRISPR/Cas9 genome editing system has revolutionized the ability to manipulate, detect, and document specific DNA and RNA sequences in cells of several species. Among other things, it has emerged to understand better and correct pathologies of genomic origin [16,17].

In our previous study in a bovine model, we edited the *TFAM* gene due to the possibility of this gene being the main regulator of mtDNA replication. This new bovine fibroblast lineage edited with heterozygosity of the *TFAM* gene provided data that this gene has a direct action on bovine mtDNA [18].

Considering the extreme importance of *TFAM* previously contextualized, we generated edited cells that present heterozygosity of the *TFAM* gene in a bovine model and proposed to characterize them to evaluate their influence on the maintenance of mtDNA and possible alterations found after the disruption of this gene. In this study, we were able to keep the edited cells viable in culture. We validated that the *TFAM* gene edition is directly linked to the decrease in the number of mitochondrial copies and mitochondrial membrane potential, confirming its direct action in the maintenance and integrity of mtDNA.

## Materials and methods

This study was approved by the Research Ethics Committee (Approval No. 5828250215) of the Faculty of Animal Science and Food Engineering, University of São Paulo, Brazil. All experiments were done in triplicate with 3 edited clones and as control 1 non-edited clone (fibroblasts without TFAM edition).

### Cell line, CRISPR design, and transfection

Bovine fibroblasts used in this study were derived from a skin biopsy, CRISPR design, and Transfection were performed as shown in [18].

### Cell viability

To test the cells' viability, they were sorted into three groups at densities of 1x10^6^ cell/cm^2^ and frozen. After one freeze-thaw round, the cells were stained with trypan blue (1:1; Sigma- catalog number T10282) to assess cell viability. The cells were frozen for 24 hours at -80°C using Nalgene Mr. Frosty (Sigma/reference number- C1562-1EA) freezing protocol and were subsequently transferred and stored in liquid nitrogen. After freezing and thawing, the cells were counted with a hemocytometer using a Neubauer chamber (GridOptik / reference number—OG-500).

### Doubling time

Cells were plated in passage 4 and counted using a Neubauer chamber (GridOptik/ reference number- OG-500). Then, the cells (3x10^4^) were plated on 35- mm plates and maintained in incubators at 37°C. The cells were replated every 3 days at the same density. The doubling time was calculated using the formula Ct/Cd, where Ct represents the culture time between passage n and passage n þ 1, and Cd represents the cell doubling. Cell doubling was calculated using the formula: Cd ¼ ln (nf/ni)/ ln2, where nf represents the harvested cells, and ni represents the seeded cells [19].

### Determination of mtDNA copy number

The cells in different cellular passages were subjected to genomic DNA extraction using the QIAmp DNA micro kit (Qiagen, 56304), according to the manufacture’s protocol. The DNA was quantified by spectrophotometry (NanoDrop 2000, Thermo Scientific, Waltham, MA, USA) and frozen at -80°C. mtDNA quantification was then performed on a real-time PCR thermocycler (Applied Biosystems, 7500 Fast Real-Time PCR System, Foster City, CA, USA) using a commercial assay system (SYBR® Green PCR Master Mix; Life Technologies) following the manufacturer instructions. The samples were analyzed in duplicate using the endogenous beta-actin gene (ACTB) as control and primers listed in Table 1. The mtDNA copy number was estimated at each cellular passage [20].

**Table 1:** Primers used for relative quantification of the target gene (mtDNA) and endogenous control (ACTB).

Target gene (Genebank access)	Primer	Sequence (5’-3’)	Product
ACTB (NM_173979.3)	ACTB-f	GGCACCCAGCACAATGAAGA	67bp
	ACTB-r	GCCAATCCACACGGAGTACTT	
Mt-RNR 2 (AY526085 / AY126697)	bMT3010-f	GCCCTAGAACAGGGCTTAGT	87bp
	bMT3096-r	GGAGAGGATTTGAATCTCTGG	

### Assessment of mitochondrial membrane potential using the Mitotracker Green and Red assays

Assessment of mitochondrial membrane potential was also done by using the Mitotracker Red and Green assays. Cells were cultured in 6 well plates. Next, 0,2ul /mL of Mitotracker Green (Invitrogen^TM^- catalog number M7514) and 0,5ul/mL of Mitotracker Red (Invitrogen^TM^ -catalog number M22425) were used for cell incubation for 30 minutes. Analyses were performed by epifluorescence microscopy (Axioplan, Carl Zeiss) and evaluated by ImageJ software. The ΔΨm was determined considering the estimated red/ green fluorescence ratio with the appropriate lasers. Corrected total cell fluorescence was mensured by imageJ using integrated density, mean grey and cell area, which is related to the level of fluorescence present in the cell. To access certain functional level into the cell, surface area of the cells was meansured and compared between edited and non-edited cells [21,22].

### Statistical analysis

The data obtained from experimental procedures were analyzed using the SAS program University Edition®, with previous verification of the normality of the residues by the Kolmogorov-Smirnov test. The variables that did not meet the statistical assumptions were submitted to the logarithmic transformation [Log (X + 1). When this procedure was necessary, the original or transformed data were submitted to Analysis of Variance (p<0.05). The time and treatment effects were evaluated by the Tukey–Krammer’s test. The data from “mitochondrial DNA copies” were analyzed by Fisher’s exact test. Effects were considered significant when p<0.05.

## Results

### Cell line

**Fig 1:**
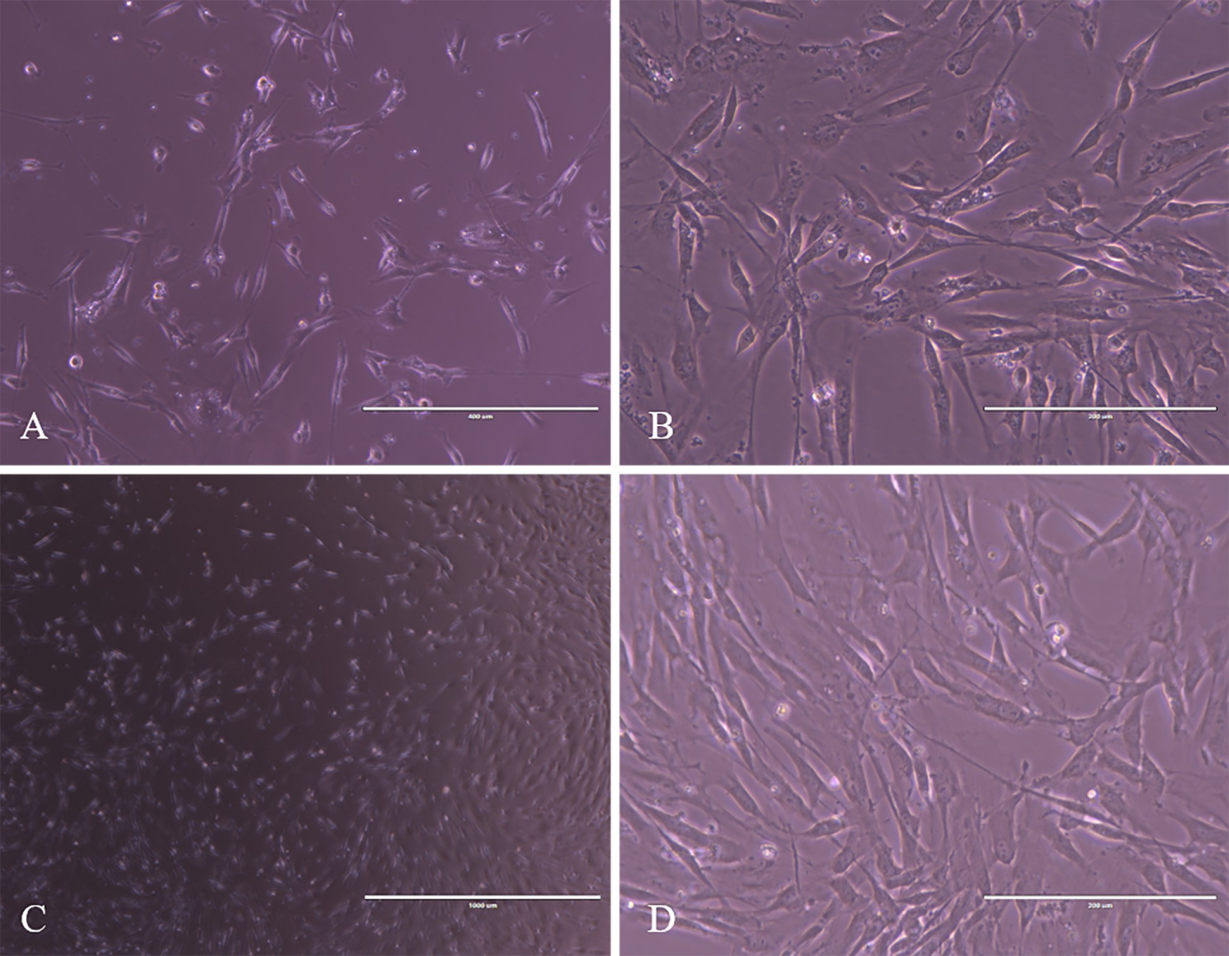
Photomicrographs of edited cells. A) cell at 24h in culture with fibroblastoid format. B) cells at 72 hours in culture note cell confluence. C) and D) observe the 80% of confluence after 6 days in culture 20X; 40X.

After cryopreservation and thawing, the cells were maintained in culture. After 24 hours in culture, the cells started to adhere to plastic and presented fibroblastoid characteristics. After 6 days in culture, the culture dish already presented 80% confluence (Fig 1). We performed successive passages, and the cells remained in culture for 30 days, after this period, they showed suggestive cellular death since we were able to observe a reduced cell size. The *in vitro* maintenance of the edited cells was achieved through the supplementation with uridine and pyruvate in the culture medium, the lack of supplementation resulted in cell death.

### Cell viability

The cell viability analysis showed a total of 84.34% of living non-edited cells and 15.66% of dead cells. Regarding the edited cells, the percentage was 78.2% of living cells and 21.8% of dead cells (Table 2), showing that the viability was somewhat lower in the edited cells when compared to control (non-edited cells). Even though the viability rate was lower, the edited cells were viable in culture after thawing.

**Table 2:** Average of the viability of the edited and non-edited cells.

	Number of cells	Live cells (total) *	Alive (%)	Dead (%)
Non-edited cells	10x10^4^	8.43x10^4^±5.03 x10^4^	84.33	15.76
Edited cells	10x10^4^	7.82x10^4^±3.76 x10^4^	78.20	21.80

* Significant differences between groups (Fisher’s exact test; p<0.05).

### Growth curve and doubling time

**Fig 2:**
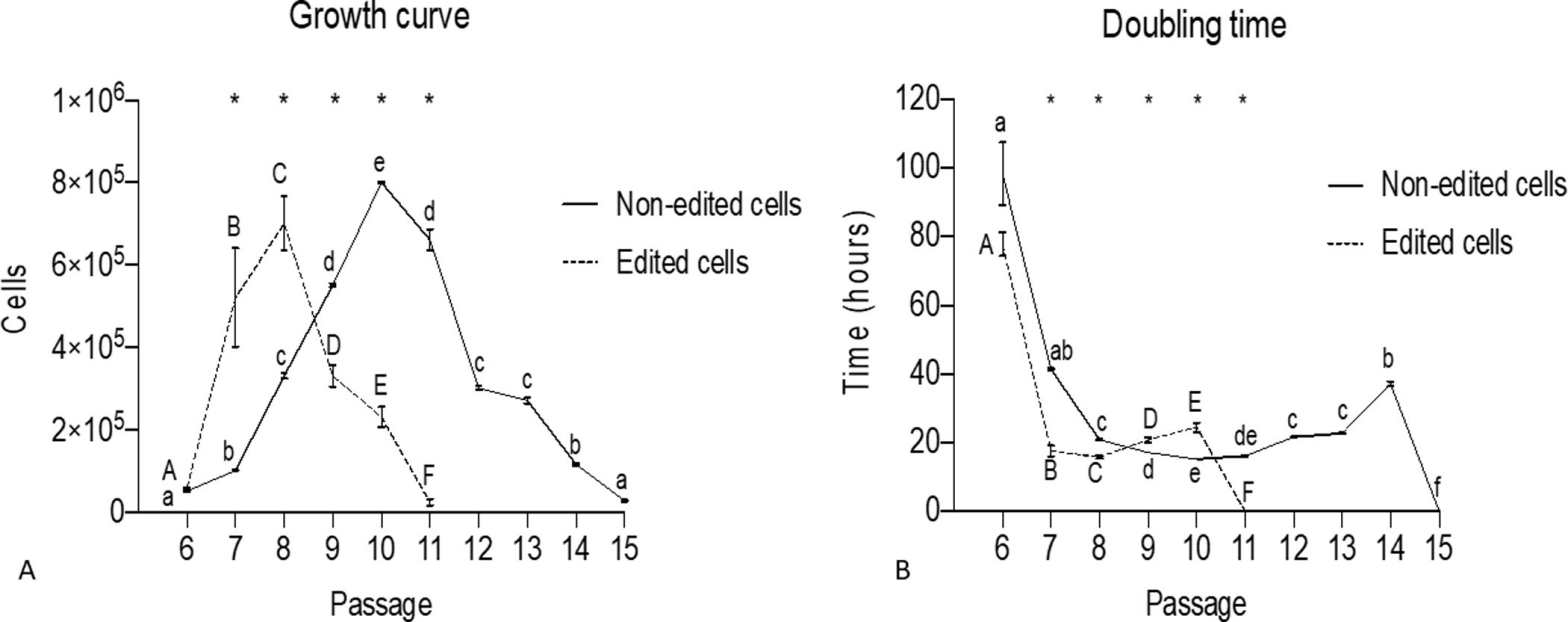
Growth curve and cell doubling time for non–edited and edited cells. a-f Different lowercase letters indicate significant differences between non-edited cells (p<0.05). A-F Different lowercase letters indicate significant differences between non-gene-edited cells (p<0.05). * Significant differences between edition within passage (p<0.05).

The non-edited and edited cells after thawing were expanded in culture. It was possible to observe that the non-edited cells were maintained until passage 15, and were more proliferative in culture at P11 (reaching a peak as shown in the figure). The edited cells were maintained in culture until passage 11 and were more proliferative in passage 8 (growth curve peak). In both, the cells doubling time increased with each new passage, meaning that the proliferation rate was reduced, showing that the genomic edition did not affect theirs *in vitro* maintenance (Fig 2).

### Determination of mtDNA copy number

**Fig 3:**
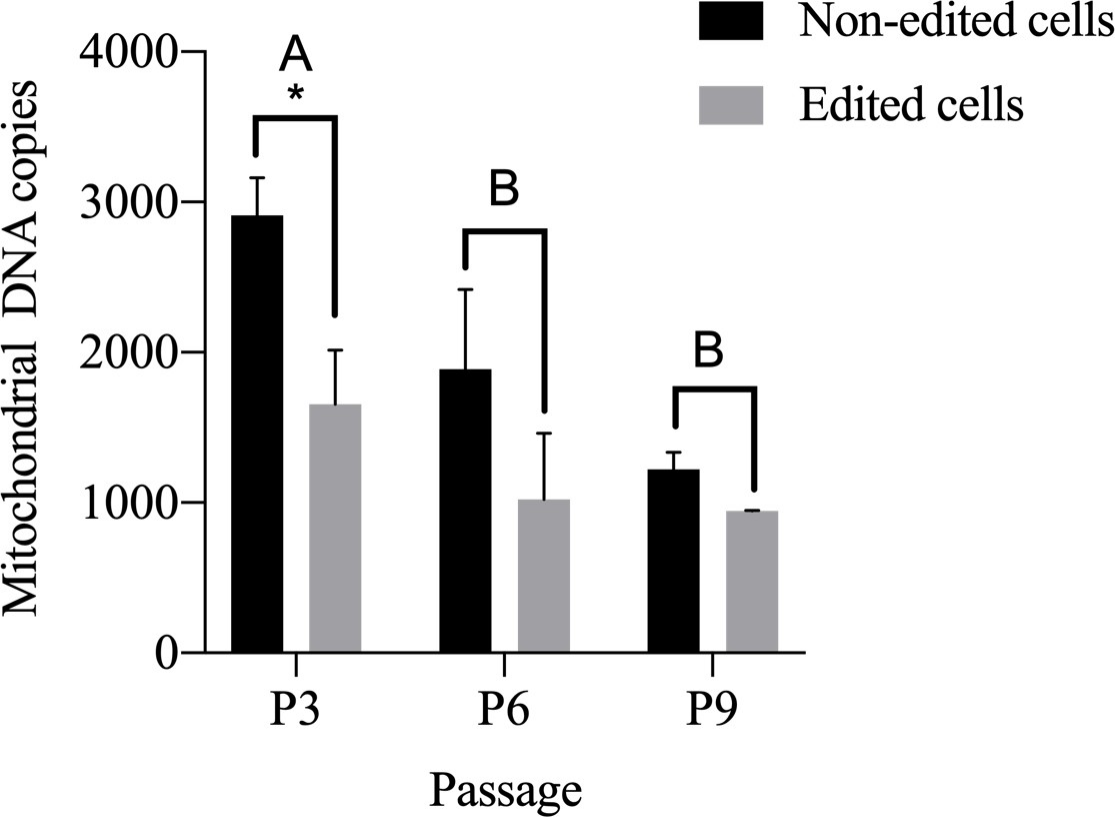
mtDNA copies per cell in different cell passages. Non-edited cells (control) and edited cells. Note non-edited cells in passage 3 with 2.912 copies and edited cells with 1.655 mitochondrial DNA copies; in passage 6 note non-edited cells with 1.889 copies and edited cells with 1.020 copies. In passage 9 observe a greater decline. Note non-edited cells with 1.222 copies and edited cells with 945 copies. A-B Different capital letters indicate significant differences between the passages, regardless of the gene edition (p <0.05). * Differences between gene edition within the passage (p <0.05).

Regarding the determination of the mitochondrial DNA copies, we observed a decrease in the mtDNA number of edited cells after successive culture passages when compared to the non-edited cells (control). In passage 3, we noticed 2.912 copies in the non-edited cells versus 1.655 in the edited clones [18]. In passage 6, the non-edited cells had 1.889 copies versus 1.020 in the edited cells. In a later passage, P9, we noticed a decrease from 1.222 copies of the non-edited cells to 945 copies of the edited cells. This shows that the mitochondrial DNA copy number decreased after each passage and compared to the control. There was a significant difference between the passages regardless of editing (p<0.05), showing that the longer the time in culture, the lower the number of mitochondrial DNA copies (Fig 3).

When comparing the edited versus non-edited cells regardless of cell passage, we noticed that the edition decreased the number of mitochondrial DNA copies, with a significant difference between edited and non-edited cells (p<0.05).

Evaluating the gene edition and cell passages together, we noticed that passage 3 is significantly different from passages 6 and 9 (p<0.05). On the other hand, passages 6 and 9 did not differ among them. These results reveal that the CRISPR/Cas9 editing was efficient. Even though only one allele was edited, it was enough to present a significant difference in the mtDNA copy number during the successive passages.

## Assessment of mitochondrial membrane potential using the Mitotracker Green and Red assays

### Epifluorescence microscopy

**Fig 4:**
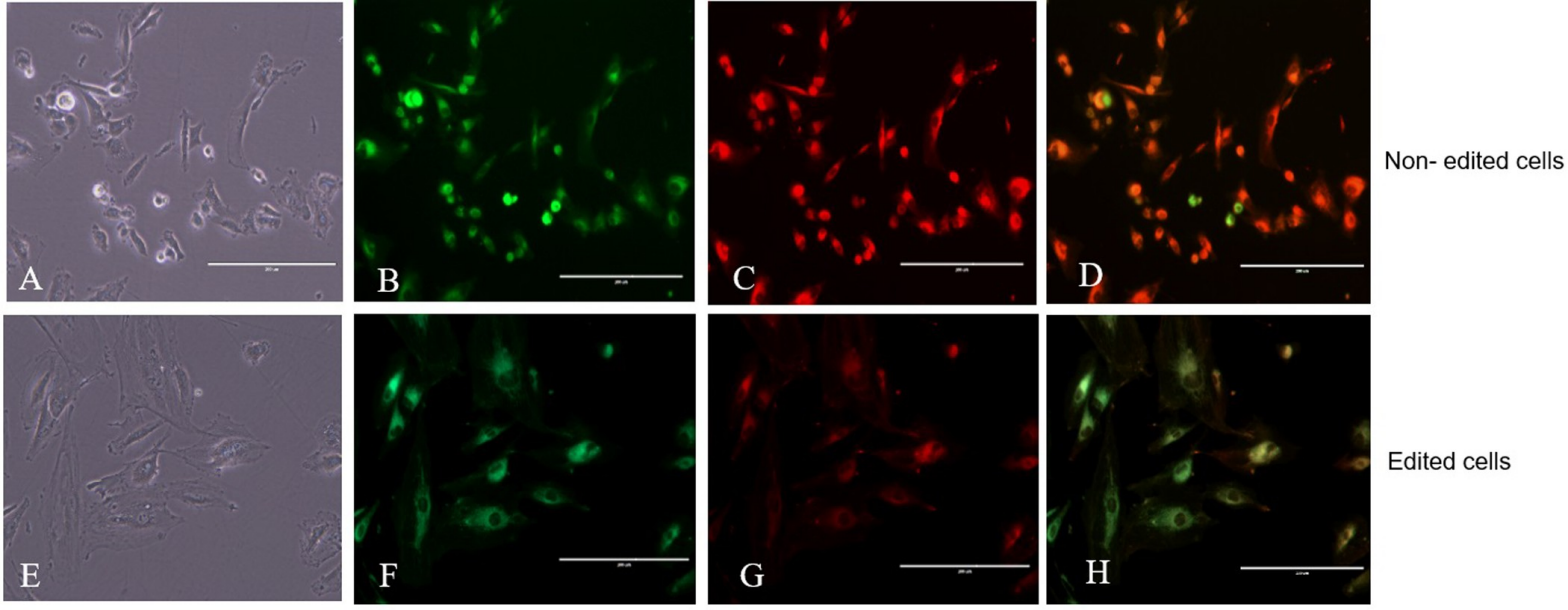
Photomicrograph of epifluorescence with Mitotracker Green and Red markers. Analysis of the mitochondrial membrane potential of edited versus non-edited cells. In A and E control, B-F Mitotracker Green fluorescence, C-G Mitotracker Red fluorescence, and D-H merged.

**Fig 5:**
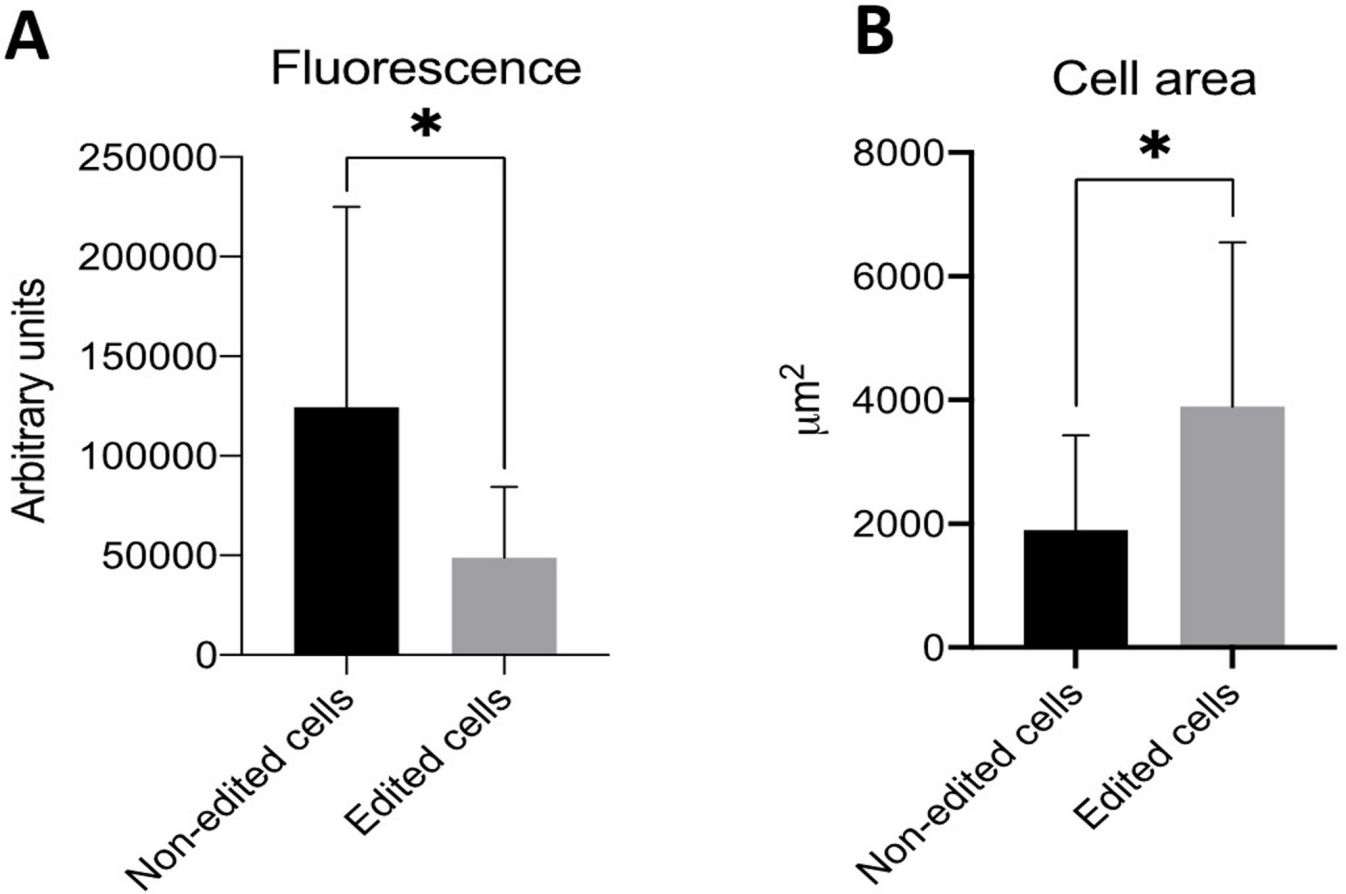
Comparation of Mitotracker red expression and cell surface area in edited and non-edited cells. (A) Red fluorescence intensity in edited and non-edited cells. (B) Cell surface area in edited and non-edited cells.

The edited cells showed a classic fibroblast appearance in culture, proving that the supplemented uridine and pyruvate were capable of supporting normal cell growth. The cell staining with Mitotracker Green and Red clearly shows a reduced of green and red fluorescence in the edited cells (Fig 4).The corrected total cell fluorescence of red was calculated by ImageJ software, edited cells showed 51851.05 (in average) intensity of red fluorescence and non- edited cell showed 121241.18 (in average) intensity of red fluorescence (Fig 5A). When compared edited cells expressed 57.24% (in average) less red fluorescence intensity than non-edited. When cell surface area was compared, non-edited cells were smaller than edited cells (Fig 5B). Lastly, cell size and fluorescence expression (mtDNA) presented a positive but not perfect linear relation (R^2^ = 0.2) (S1 Fig).

## Discussion

Because *TFAM* binds to specific regions of mtDNA, acting in replication, transcription of mtDNA and performs functions in the compaction of this genome, exerts specific functions of organelle maintenance we proposed to edit this gene by CRISPR/Cas9 and evaluate the cellular characteristics after editing in bovine fibroblasts, to observe their influence on the maintenance of mtDNA. The results after this process were seven heterozygous mutant clones [18]. The bovine was chosen as a model due to its phylogenetic similarity to humans [23].

The *TFAM* gene has been disrupted before in mice and chickens with homozygous gene mutation showing embryonic lethality and mtDNA depletion [24,25].

Our study performed the edition of the *TFAM* gene in bovine fibroblasts and obtained heterozygous clones [18]. The interruption of this gene can lead to lethality, so we followed the protocol of *in vitro* cell maintenance through supplementation with uridine and pyruvate, as previously used in bovine fibroblasts in a study showing the treatment of these cells with ethidium bromide to deplete the mtDNA and evaluate the effects on the mtDNA copy number and their action on cell metabolism [26].

After thawing, we expanded the edited cells in culture and performed their characterization. We believe that the *in vitro* maintenance of the edited cells is due to the supplementation mentioned above because according to the data, cell viability was 78.54% of living cells in the edited clones versus 84.34% of the non-edited cells. We believe that the editing did not affect cell viability and proliferation since the Doubling time results were positive, showing an increase in each successive passage, asserting again that the editing did not affect the cell proliferation and growth rates until the 11th passage in culture.

When we compare the growth curve of edited versus non-edited cells, the edited cells have a shorter life span. This more limited life span may be related to a decreased number of mitochondrial DNA copies.

The mitochondrial biogenesis coordinates the mitochondrial population according to cell energy demands [27]. TFAM participates in the regulation of mitochondrial biogenesis together with mtDNA. A study with bovine adipose-tissue-derived mesenchymal cells performed mtDNA depletion by treating the cells with ethidium bromide. Similarly to our study, they determined mtDNA copy number and found that it decreased as successive days in culture went by, reaching a 90% depletion rate after 13 days of EtBr treatment [28]. In a study with HeLa cells, mtDNA was also depleted with EtBr. Besides the successful depletion, authors also reported reduced TFAM and RNA polymerase levels, showing a complex interaction between mtDNA copy number and other necessary factors for mitochondrial transcription (such as TFAM and RNA polymerase) [29]. Other studies have shown that the evaluation of the mitochondrial DNA copies has been correlated to *TFAM* function, showing that when *TFAM* varies, there's a direct effect in the mtDNA copies. In embryonic development, a higher mtDNA copy number is essential. *TFAM* is believed to be the regulator of mtDNA because this gene is critical in the mitochondrial replication process [5].

It was also shown that over-expression or low expression of *TFAM* could lead to a decrease in the number of mtDNA copies [25].

In a study with human fibroblasts, the authors showed a significant increase in mtDNA copy number at late passages in culture of various diploid human cells [30]. These results suggest that the mtDNA copy number can suffer a feedback response compensating for defective mitochondria having its respiratory chain impaired or mutated mtDNA [31]. One crucial factor in aging is the accumulation of DNA damage over time, and the mtDNA has been considered a significant target of aging-associated mutation accumulation. In a different study, mtDNA heteroplasmy incidence was shown to increase with age, and at the same time, a decrease of mtDNA copy number was observed, which is similar to our findings [32]. In bovine adipose-tissue-derived mesenchymal cells with mtDNA depleted by Ethidium Bromide, the control cells with no EtBr treatment showed a decline in mtDNA copy number after some time in culture [28].

When performing the mitochondrial copy number in different cell passages, we used the already published data of passage 3 [18] as a comparison. We also confirmed this decrease in mtDNA by the reduced *in vitro* lifespan referring to cell passages and in comparison to the control, also showing that regardless of edition, the mitochondrial DNA copy number decreases with each *in vitro* passage.

When we compared the non-edited with the edited cells, we also found a significant difference regardless of cellular passage. With this, we affirm that *TFAM* disruption may affect the number of mitochondrial DNA copies. Similar results were demonstrated in a heterozygous knockout of *TFAM* in mice, highlighting the reduction of mtDNA copies in approximately 50% [24].

A study that claims to be the first to describe a pathology caused by changes in *TFAM* function showed its correlation with mitochondrial diseases. The pathology was described in two siblings and consists of a hepatocerebral syndrome caused by mtDNA depletion, which in turn is related to a specific mutation present in the family, causing neonatal liver failure, besides other symptoms, followed by death in a few months. Through exome sequencing of the patients, the team reported a homozygotic variation (c.533CNT; p. Pro178Leu) in the *TFAM* gene, a variation that may have caused a loss of protein binding capabilities in mtDNA, consequently causing a decrease in its replication, amount, and compaction in nucleoids. By analyzing the *TFAM* gene expression and the number of mtDNA copies by qPCR in the patients' fibroblasts, among other tests, they were able to effectively relate the symptoms of the syndrome to the mtDNA depletion of the affected siblings caused by the *TFAM* mutation, showing once again the importance of the integrity of this gene and its polypeptide for correct mitochondrial function [33].

Other human studies have been conducted. A British team carried out a genomic study with almost 7.000 patients whose conclusion once again pointed to this relationship between *TFAM*-related nuclear control and the number of mtDNA copies [34].

Other models also correlated the TFAM gene regulation to mitochondrial diseases by showing that the overexpression of *TFAM* generates a protective effect on cell function in several disease models, such as type II diabetes, heart attack, and heart failure [14]. In another study, the effect of increased mtDNA copy number on cardioprotection in mice was examined. They observed that the overexpression of *TFAM* could increase the mtDNA copy number and facilitate cardioprotection associated with limited mitochondrial oxidative stress [35].

Recently, a group of researchers identified a homozygous predicted pathogenic missense mutation in TFAM [NM_003201.3: c.694C>T,NP_003192.1: p.(Arg232Cys)]. Their data suggest that pathogenic TFAM variants and consequently, defects in mtDNA maintenance can be fatal (early-onset liver disease) and cause some rare genetic disorders, like Perrault syndrome [36].

Regarding the mitochondrial membrane potential, in our findings, both edited and non-edited cells presented positive markings for Mitotracker Green, thus being viable in culture. The Mitotracker Red is only emitted in mitochondria with high mitochondrial activity. Our findings showed that a lower red color in the edited cells showed less mitochondrial activity than non-edited cells (control). Edited cells showed 57.24% (in average) less red fluorescence intensity than non-edited cells, which can be related to the reduction of mtDNA. When cell surface area was compared, non-edited cells were smaller than edited cells, giving an indication of certain cell response due to metabolic changes related to mtDNA reduction [37].

Still, concerning mtDNA disruptions, a study developed a specific Cas that reaches the mitochondrial DNA, and after modifying HEK-293T cells with sgRNA targeting *Cox1* and *Cox3* genes they showed, by Mitotracker Red assay, that there was a decrease in the mitochondrial membrane potential compared to the non-edited control, as well as a decrease in cell proliferation in culture [38], findings similar to ours.

In other research LRPPRC (leucine-rich pentatricopeptide repeat-containing) depletion by activating induced autophagy, showing that LRPPRC serves as a checkpoint protein for initiation of baseline autophagy levels. LRPPRC was silenced by siRNA suppressing the gene expression, which led to the reduction of Mitotracker Red signals representing mitochondrial membrane potentials. In our study, the low mitochondrial activity shows us that *TFAM* editing has affected both the number of mtDNA copies mentioned above, as well as the mitochondrial activity [39].

The regulation of mtDNA copy number is still poorly understood. The TFAM gene is a candidate to be investigated because many studies point this gene as essential to regulate mtDNA copy number. In our study, we showed that the gene edition of TFAM affects mtDNA by decreasing its copy number and consequently altering mitochondrial activity, confirming the TFAM role in mitochondrial maintenance and function.

## Supporting information

S1 Fig Linear relation between cell size and fluorescence expression (p<0.05). (TIF)

## References

[1] Gray MW, Burger G. Lang B.F. Mitochondrial evolution. Science, 283: 1476–1481, 1999 10.1126/science.283.5407.1476 . 10066161

[2] Ernster L, Schatz G. Mitochondria: A historical review. J Cell Biol, 91: 227–255, 1981 10.1083/jcb.91.1.227. 7033239 PMC2112799

[3] Gustafsson CM, Falkenberg M, Larsson NG. Maintenance and expression of mammalian mitochondrial DNA. Annu Rev Biochem. 2016; 85:133–160. 10.1146/annurev-biochem-060815-014402 . 27023847

[4] Copeland WC, Longley MJ. Mitochondrial genome maintenance in health and disease. DNA repair. 2014; 19: 190–198. 10.1016/j.dnarep.2014.03.010 . 24780559 PMC4075028

[5] Ekstrand MI, Falkenberg M, Rantanen A, Park CB, Gaspari M, Hultenby K, et al Mitochondrial transcription factor A regulates mtDNA copy number in mammals. Hum Mol Genet. 2004; 13 (9): 935–944. 10.1093/hmg/ddh109 15016765

[6] Kukat C, Davies KM, Wurm CA, Spahr H, Bonekamp NA, Kühl I, et al Cross-strand binding of TFAM to a single mtDNA molecule forms the mitochondrial nucleoid. Proc Natl Acad Sci. 2015; 112 (36): 11288–11293. 10.1073/pnas.1512131112 26305956 PMC4568684

[7] Marín-Garcia J. Mitochondrial DNA repair: a novel therapeutic target for heart failure. Heart Fail Rev. 2016; 21 (5): 475–487. 10.1007/s10741-016-9543-x 26940911

[8] Cline S. D. Mitochondrial DNA damage and its consequences for mitochondrial gene expression. BBA-Gene Regul Mech. 2012; 1819 (9–10): 979–991. 10.1016/j.bbagrm.2012.06.002 . 22728831 PMC3412069

[9] Silva ECB. Espécies reativas de oxigênio e nitrogênio: produção e efeitos sobre a integridade estrutural e funcional dos espermatozoides. Ciência Veterinária.2010; 13 (1/2/3): 9–16.

[10] Chandrasekaran K.; Anjaneyulu M.; Choi J.; Kumar P.; Salimian M.; Ho C. Y. et al Role of mitochondria in diabetic peripheral neuropathy: Influencing the NAD+-dependent SIRT1–PGC-1α–TFAM pathway. Int Rev Neurobiol. 2019; 145:177–209. 10.1016/bs.irn.2019.04.002 . 31208524 PMC6590704

[11] Johri A.; Chandra A.; Flint Beal M. PGC-1α, mitochondrial dysfunction, and Huntington's disease. Free Radic Biol Med. 2013; 62:37–46. 10.1016/j.freeradbiomed.2013.04.016 23602910 PMC3722269

[12] Selfridge JE, E L, Lu J, Swerdlow RH. Role of mitochondrial homeostasis and dynamics in Alzheimer's disease. Neurobiol Dis. 2013; 51:3–12. 10.1016/j.nbd.2011.12.057 22266017 PMC3337963

[13] Uittenbogaard M, Chiaramello A. Mitochondrial biogenesis: A therapeutic target for neurodevelopmental disorders and neurodegenerative diseases. Curr Pharm Des. 2014; 20(35):5574–5593. 10.2174/1381612820666140305224906 24606804 PMC4823001

[14] Kang I, Chu CT, Kaufman BA. The mitochondrial transcription factor TFAM in neurodegeneration: Emerging evidence and mechanisms. FEBS Lett. 2018; 592 (5):793–811. 10.1002/1873-3468.12989 29364506 PMC5851836

[15] Rubinsztein DC. The roles of intracellular protein-degradation pathways in neurodegeneration. Nature.2006; 443: 780–786. 10.1038/nature05291 17051204

[16] Doudna JA, Charpentier E. The new frontier of genome engineering with CRISPR-Cas9. Science. 2014; 346 (6213): 1258096 10.1126/science.1258096 25430774

[17] Pickar-Oliver A, Gersbach CA. The next generation of CRISPR–Cas technologies and applications. Nat Rev Mol Cell Bio. 2019; 20 (8): 490–507. 10.1038/s41580-019-0131-5 31147612 PMC7079207

[18] Oliveira VC, Moreira GSA, Bressan FF, Mariano Junior CG, Roballo KCS, Charpentier M, et al Edition of TFAM gene by CRISPR/Cas9 technology in bovine model. PLoS One. 2019; 14 (3):1–14. 10.1371/journal.pone.0213376 30845180 PMC6405117

[19] Raoufi MF, Tajik P, Dehghan MM, Eini F, Barin A. Isolation and differentiation of mesenchymal stem cells from bovine. Reprod Domest Anim. 2011 46 (1):95–99. 10.1111/j.1439-0531.2010.01594.x 20345587

[20] Nicklas JA.; Brooks EM.; Hunter TC.; Single R.; Branda RF. Development of a quantitative PCR (TaqMan) assay for relative mitochondrial DNA copy number and the common mitochondrial DNA deletion in the rat. Environ Mol Mutagen. 2004; 44 (4): 313–320. 10.1002/em.20050 15476199

[21] Roballo KCS, Bushman J. Evaluation of the host immune response and functional recovery in peripheral nerve autografts and allografts. Transpl. Immunol. 2019; 53: 61–71. 10.1016/j.trim.2019.01.003 30735701

[22] McCloy RA, Rogers S, Caldon CE, Lorca T, Castro A, Burgess, A. Partial inhibition of Cdk1 in G 2 phase overrides the SAC and decouples mitotic events. Cell Cycle. 2014; 13:1400–1412. 10.4161/cc.28401 24626186 PMC4050138

[23] Elsik CG, Tellam RL, Worley KC. The genome sequence of Taurine cattle: A window to ruminant biology and evolution. Science. 2009; 324: 522–527. 10.1126/science.1169588 19390049 PMC2943200

[24] Larsson NG, Wang J, Wilhelmsson H, Oldfors A, Rustin P, Lewandoski M, et al Mitochondrial transcription factor A is necessary for mtDNA maintenance and embryogenesis in mice. Nat. Genet. 1998; 18 (3): 231–36. 10.1038/ng0398-231 9500544

[25] Kang D, Hamasaki N. Mitochondrial Transcription Factor A in the Maintenance of Mitochondrial DNA Overview of Its Multiple Roles Annals of the New York Academy of Sciences. Ann Ny Acad Sci. 2005; 1042: 101–108. 10.1196/annals.1338.010 15965051

[26] Chiaratti MR, Meirelles FV. Increase in mitochondrial DNA quantity and impairment of oxidative phosphorylation in bovine fibroblast cells treated with ethidium bromide for 15 passages in culture. Genet. Mol. Res. 2006; 5 (1): 55–62. . 16755497

[27] Moraes CT. What regulates mitochondrial DNA copy number in animal cells? Trends Genet. 2001; 17: 199–205. 10.1016/s0168-9525(01)02238-7 . 11275325

[28] Pessoa LVF, Bressan FF, Chiaratti MR, Pires PRL, Perecin D, Smith LC, et al Mitochondrial DNA dynamics during *in vitro* culture and pluripotency induction of a bovine Rho0 cell line. Genet. Mol. Res. 2015; 14 (4): 14093–14104. 10.4238/2015.October.29.29 . 26535724

[29] Seidel-Rogol BL, Shadel GS. Modulation of mitochondrial transcription in response to mtDNA depletion and repletion in HeLa cells. Nucleic Acids Res. 2002; 30: 1929–1934. 10.1093/nar/30.9.1929 . 11972329 PMC113853

[30] Shmookler Reis RJ, Goldstein S. Mitochondrial DNA in mortal and immortal human cells. J Biol Chem.1983; 258: 90789085. . 6307991

[31] Moskalev AA, Shaposhnikov MV, Plyusnina EN, Zhavoronkov A, Budovsky A, Yanai H, et al The role of DNA damage and repair in aging through the prism of Koch-like criteria. Ageing Res Rev. 2013;12(2):661–684. 10.1016/j.arr.2012.02.001 . 22353384

[32] Sondheimer N, Glatz CE, Tirone JE, Deardorff MA, Krieger AM, Hakonarson. Neutral mitochondrial heteroplasmy and the influence of aging. Hum Mol Genet. 2011; 20(8):1653–9. 10.1093/hmg/ddr043 . 21296868 PMC3063991

[33] Stiles AR, Simon MT, Stover A, Eftekharian S, Khanlou N, Wang HL, et al Mutations in TFAM, encoding mitochondrial transcription factor A, cause neonatal liver failure associated with mtDNA depletion. Mol Genetic Metab.2016;11: 91–9, 2016. 10.1016/j.ymgme.2016.07.001 27448789

[34] Guyatt AL, Brennan RR., Burrows K, Guthrie P, Ascione R, Ring SM, et al A genome-wide association study of mitochondrial DNA copy number in two population-based cohorts. Human genomics. 2019; 13 (1): 6 10.1186/s40246-018-0190-2 30704525 PMC6357493

[35] Ikeda M, Ide T, Fujino T, Arai S, Saku K, Kakino T, et al Overexpression of TFAM or twinkle increases mtDNA copy number and facilitates cardioprotection associated with limited mitochondrial oxidative stress. PLos One. 2015; 10 (3): e0119687 10.1371/journal.pone.0119687 25822152 PMC4379048

[36] Tucker EJ, Rius R, Jaillard S, et al Genomic sequencing highlights the diverse molecular causes of Perrault syndrome: a peroxisomal disorder (PEX6), metabolic disorders (CLPP, GGPS1), and mtDNA maintenance/translation disorders (LARS2, TFAM) Hum Genet. 2020;10.1007/s00439-020-02176-w. 10.1007/s00439-020-02176-w . 32399598

[37] DeBerardinis RJ, and Thompson CB. Cellular metabolism and disease: what do metabolic outliers teach us? Cell. 2012148, 1132–1144. 10.1016/j.cell.2012.02.032 . 22424225 PMC3337773

[38] Jo A, Ham S, Lee GH, Lee YI, Kim S, Lee YS, et al Efficient mitochondrial genome editing by CRISPR/Cas9. Bio Med Res international. 2015; 2015: 10. 10.1155/2015/305716 PMC4581504 26448933

[39] Zou J, Yue F, Jiang X, Li W, Yi J, Liu L. Mitochondrion-associated protein LRPPRC suppresses the initiation of basal levels of autophagy via enhancing Bcl-2 stability. Biochemical. 2013; 454(3): 447–457. 10.1042/BJ20130306 23822101 PMC3778712

