# Supplementary figures and images for: Characterization of post-edited cells modified in the TFAM gene by CRISPR/Cas9 technology in the bovine model
Source: PLoS One. 2020 Jul 10;15(7):e0235856. doi: 10.1371/journal.pone.0235856 (PMC7351154; doi:10.1371/journal.pone.0235856)

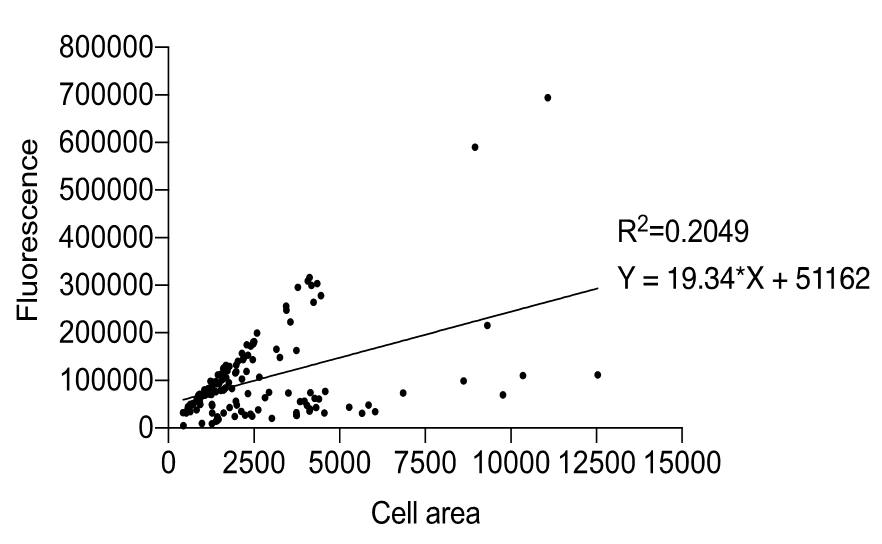

Supplement: S1 Fig — (TIF) [file pone.0235856.s001.tif]
